# Atypical Presentation of Thrombotic Thrombocytopenic Purpura (TTP) as an Initial Manifestation of Systemic Lupus Erythematosus (SLE) in a Young Male

**DOI:** 10.7759/cureus.67567

**Published:** 2024-08-23

**Authors:** Kristen Pitts, Andres D Parga, Damian Casadesus, Stephen Symes

**Affiliations:** 1 Medicine, American University of the Caribbean, Cupecoy, SXM; 2 Internal Medicine, Jackson Memorial Hospital, Miami, USA; 3 Infectious Diseases, University of Miami Miller School of Medicine, Miami, USA

**Keywords:** plasma exchange therapy, hydroxychloroquine treatment, microvascular thrombosis, autoimmune diseases, adamts13, systemic lupus erythematosus, thrombotic thrombocytopenic purpura

## Abstract

Thrombotic thrombocytopenic purpura (TTP) is a rare but life-threatening hematologic disorder characterized by thrombocytopenia, microangiopathic hemolytic anemia, neurologic symptoms, renal impairment, and fever. The etiology of TTP often involves a severe deficiency in ADAMTS13 activity, resulting in the accumulation of ultra-large von Willebrand factor multimers and subsequent microvascular thrombosis. Systemic lupus erythematosus (SLE) is a chronic autoimmune disease that can affect multiple organ systems, and although the initial presentation of SLE with TTP is rare, it necessitates a comprehensive diagnostic and therapeutic approach. We present a case of a 27-year-old male with no significant past medical history who developed altered mental status, headache, and right-sided numbness, leading to the diagnosis of TTP and subsequent detection of SLE.

## Introduction

Thrombotic thrombocytopenic purpura (TTP) is a hematological emergency characterized by a pentad of symptoms: thrombocytopenia, microangiopathic hemolytic anemia, neurological abnormalities, renal dysfunction, and fever. The condition is often associated with severe deficiency in ADAMTS13, a metalloprotease that cleaves von Willebrand factor (vWF) multimers. This deficiency leads to the accumulation of ultra-large vWF multimers, promoting platelet aggregation and microvascular thrombosis [1]. Systemic lupus erythematosus (SLE) is a systemic autoimmune disorder characterized by the presence of autoantibodies and the potential to affect multiple organ systems. The coexistence of TTP with SLE is rare in itself. Furthermore, TTP being the initial presentation of SLE is even more exceedingly uncommon [2]. This case report highlights the diagnostic and therapeutic challenges associated with such an atypical presentation, emphasizing the importance of prompt recognition and intervention.

## Case presentation

A 27-year-old male with no significant medical history arrived at the ED from an outside facility, presenting with altered mental status, headache, and right-sided numbness of one day's duration. Initial evaluation at the prior facility led to a stroke alert, and imaging studies, including CT/CTA of the head and MRI of the brain, were negative for acute cerebrovascular accident (Table 1). The patient was noted to be febrile with scattered petechiae on his arms and back.

**Table 1:** Initial imaging and clinical findings

Investigation	Result	Findings
CT/CTA of Head	Negative	No acute cerebrovascular accident
MRI of Brain	Negative	No acute lesions identified
Physical Examination	Febrile, petechiae	Scattered petechiae on arms and back

Laboratory investigations revealed a severely decreased platelet count and hemoglobin (Table 2). These findings, in conjunction with the clinical presentation, raised suspicion for TTP, prompting transfer to our institution for further management.

**Table 2:** Laboratory results at presentation

Laboratory Test	Result	Reference Range
Hemoglobin	7.8 g/dL	13.5 - 17.5 g/dL
Hematocrit	23.4%	41.0 - 53.0%
White Blood Cell Count	6.8 x 10^3/µL	4.5 - 11.0 x 10^3/µL
Platelet Count	11,000/µL	150,000 - 450,000/µL
ADAMTS13 Activity	14%	50 - 160%
Total Bilirubin	2.1 mg/dL	0.1 - 1.2 mg/dL
Reticulocyte Count	10.1%	0.5 - 2.5%
Fibrinogen	259 mg/dL	200 - 400 mg/dL
D-dimer	4.89 µg/mL	<0.50 µg/mL
Creatinine	1.2 mg/dL	0.6 to 1.2 mg/dL

Upon arrival, the patient exhibited the classic pentad of TTP symptoms: fever, neurological manifestations, acute kidney injury (AKI), anemia, and thrombocytopenia. On physical examination, the patient exhibited marked pallor, weakness, and a thin habitus with widespread petechiae on the arms and back. These petechiae, consistent with the microvascular thrombosis seen in TTP were characterized by numerous non-blanching, erythematous to purpuric macules, ranging from 1 to 3 mm in diameter. The lesions were particularly prominent in areas of friction and pressure, suggesting ongoing platelet consumption and endothelial damage. The skin also showed evidence of ecchymoses in the dependent regions, further highlighting the severe thrombocytopenia and associated coagulopathy.

ADAMTS13 activity was markedly reduced, consistent with TTP. A peripheral blood smear revealed moderate schistocytes, supporting the diagnosis of microangiopathic hemolytic anemia. The PLASMIC score assessment indicated a high risk for severe ADAMTS13 deficiency, correlating with a 72% likelihood of TTP [3]. The patient was admitted to the Medical Intensive Care Unit (MICU), where a central venous catheter (CVC) was placed for therapeutic plasma exchange, initiated immediately in conjunction with high-dose corticosteroid therapy (prednisone 70 mg daily). During the hospital course, the patient underwent multiple plasma exchange sessions. By day six, following four sessions, his platelet count increased to over 150,000/µL. However, a subsequent decline to 80,000/µL on the following day prompted the resumption of plasma exchange and continuation of steroid therapy due to concerns about ongoing TTP.

Further serological testing revealed positive rheumatoid factor (RF), antinuclear antibody (ANA), anti-SSA, anti-SSB, anti-RNP, and anti-Smith (Sm) antibodies, along with low complement levels (C3 and C4) (Table 3). These findings were suggestive of systemic lupus erythematosus (SLE). Rheumatology was consulted, and hydroxychloroquine (Plaquenil) 200 mg daily was initiated. Following several additional plasma exchange treatments, the patient's platelet count improved to 263,000/µL, and ADAMTS13 activity increased to 19%. The patient was subsequently discharged with instructions for outpatient follow-up with hematology and rheumatology.

**Table 3:** Autoimmune serology

Test	Result
Rheumatoid Factor (RF)	Positive
Antinuclear Antibody (ANA)	Positive
Anti-SSA	Positive
Anti-SSB	Positive
Anti-RNP	Positive
Anti-Smith (Sm)	Positive
Complement C3	Low
Complement C4	Low

## Discussion

This case presents a unique and challenging dilemma in which TTP arose as the initial manifestation of SLE in a young male. TTP is a rare hematologic emergency characterized by severe ADAMTS13 deficiency, leading to the accumulation of ultra-large von Willebrand factor multimers and subsequent microvascular thrombosis. The concomitant presentation of SLE, an autoimmune disorder with multi-organ involvement, significantly complicates the diagnostic and therapeutic approach, requiring a high degree of clinical suspicion and multidisciplinary coordination.

TTP in patients with SLE is extremely rare. The overall incidence of TTP in SLE patients is unclear and has been reported to be as low as 0.5% [4]. The pathophysiological mechanisms linking these two conditions are not fully understood but are believed to involve immune dysregulation and autoantibody production, leading to endothelial damage and platelet aggregation [5]. This case exemplifies the complex interplay between autoimmune dysregulation and thrombotic microangiopathy. The identification of positive autoantibodies (anti-SSA, anti-SSB, anti-RNP, and anti-Smith) and low complement levels in our patient supports the diagnosis of SLE and suggests that autoimmune processes may have triggered the TTP episode. This dual diagnosis stresses the need for comprehensive autoantibody screening in patients presenting with TTP, as identifying an underlying autoimmune condition can significantly influence management strategies.

Early recognition and treatment of TTP are crucial to prevent severe complications such as neurological deficits and renal failure. In this case, the prompt initiation of therapeutic plasma exchange (TPE) and corticosteroid therapy was pivotal in managing the acute phase of TTP. The therapeutic goal of TTP is to rapidly reduce the circulating autoantibodies and replenish ADAMTS13 activity, thus mitigating the risk of ongoing microvascular thrombosis [6]. The transient decrease in platelet count observed during the hospital course highlights the need for vigilant monitoring and the potential requirement for prolonged or repeated TPE sessions. This fluctuation in platelet levels underscores the dynamic nature of TTP and the importance of individualized treatment protocols.

Hydroxychloroquine, an antimalarial with immunomodulatory properties, was introduced following the diagnosis of SLE. This medication is a cornerstone in the management of SLE and helps to reduce disease activity and prevent flares. The role of hydroxychloroquine in stabilizing the immune system and its beneficial effects on lipid profiles and thrombosis risk make it an essential component of long-term management for SLE patients [7]. The patient's response to TPE and corticosteroids, combined with the introduction of hydroxychloroquine, resulted in a favorable outcome, as evidenced by the improvement in platelet count and ADAMTS13 activity.

This case emphasizes the importance of considering autoimmune diseases such as SLE in the differential diagnosis of TTP, especially in young patients presenting with atypical features. The overlap in clinical and laboratory findings between TTP and SLE necessitates a comprehensive diagnostic approach, including serological testing and close collaboration between hematologists and rheumatologists. The integration of specialized care from both disciplines can facilitate timely and accurate diagnosis, ensuring that appropriate therapeutic measures are implemented promptly.

Future research should focus on elucidating the underlying mechanisms that link TTP and SLE. Understanding the role of autoantibodies, complement activation, and endothelial dysfunction in these overlapping syndromes could unveil novel therapeutic targets. Additionally, the development of more sensitive and specific diagnostic markers for TTP in the context of autoimmune diseases could enhance early detection and improve patient outcomes. Advancements in genetic and molecular profiling may also provide deeper insights into the predisposition and pathogenesis of such complex presentations, ultimately guiding the development of targeted and personalized treatment strategies [8]. By advancing our knowledge and refining our clinical practices, we can enhance the quality of life and clinical outcomes for patients affected by these challenging conditions.

## Conclusions

This case report illustrates a rare and intriguing instance of TTP as the initial manifestation of SLE, highlighting the diagnostic and therapeutic complexities associated with such presentations. The successful management of this patient was achieved through prompt recognition, immediate initiation of TPE, and corticosteroid therapy, followed by the introduction of hydroxychloroquine for SLE. This case serves as a reminder of the importance of a thorough and systematic approach to patients with hematologic emergencies, considering the possibility of underlying autoimmune disorders.

Clinicians should maintain a high index of suspicion for underlying autoimmune conditions in patients presenting with TTP, particularly when atypical features are observed. The integration of clinical findings, laboratory results, and serological testing is essential in distinguishing between isolated TTP and TTP associated with conditions such as SLE. Early and accurate diagnosis, combined with a multidisciplinary approach to treatment, is essential for optimizing outcomes in these challenging cases. The collaboration between hematologists, rheumatologists, and other specialists ensures comprehensive care and maximizes the chances of a favorable prognosis.
